# Longitudinal evaluation of the psychometric stability of the somatic symptom scale-8 (SSS-8) in a large German general population sample

**DOI:** 10.1038/s41598-026-51808-0

**Published:** 2026-05-21

**Authors:** Julia Petersen, Elmar Brähler, Nora Hettich-Damm, Rieke Baumkötter, Philipp S. Wild, Alica Hartmann, Karl J. Lackner, Jochem König, Manfred E. Beutel

**Affiliations:** 1 Department of Psychosomatic Medicine and Psychotherapy, University Medical Center of the Johannes Gutenberg-University Mainz, Zahlbacher Straße 8, 55131 Mainz, Germany; 2 Clinic and Polyclinic for Psychosomatic Medicine and Psychotherapy, Rostock University Medical Center, Rostock, Germany; 3 Preventive Cardiology and Preventive Medicine, Center for Cardiology, University Medical Center of the Johannes Gutenberg-University Mainz, Mainz, Germany; 4 Partner Site Rhine Main, German Center for Cardiovascular Research (DZHK), University Medical Center of the Johannes Gutenberg-University Mainz, Mainz, Germany; 5 Center for Thrombosis and Hemostasis (CTH), University Medical Center of the Johannes Gutenberg University Mainz, Mainz, Germany; 6 Institute of Molecular Biology (IMB), Mainz, Germany; 7 Department of Ophthalmology, University Medical Center of the Johannes Gutenberg University Mainz, Mainz, Germany; 8 Institute of Clinical Chemistry and Laboratory Medicine, University Medical Center of the Johannes Gutenberg-University Mainz, Mainz, Germany; 9 Institute of Medical Biostatistics, Epidemiology and Informatics, University Medical Center of the Johannes Gutenberg-University Mainz, Mainz, Germany

**Keywords:** Somatic Symptom Scale, psychometrics, confirmatory factor analysis, measurement invariance, SARS-CoV-2, Psychology, Public health

## Abstract

The Somatic Symptom Scale (SSS-8) is a brief 8-item tool for screening common somatic symptoms. This study is the first to examine its temporal invariance and stability across three COVID-19 pandemic time points, while also analyzing changes in SSS-8 scores in the general population and across groups (sex, age, relationship status, poverty status), and testing temporal stability and measurement invariance. Longitudinal data from three pandemic time points in a large population-based sample were used. Temporal stability was assessed using Pearson correlations, and confirmatory factor analysis with measurement invariance testing examined factor validity and temporal invariance. The SSS-8 decreased from the first to the second measurement and subsequently increased between the second and third time points. The scale demonstrated good internal consistency (α ≥ 0.78, ω ≥ 0.82) and temporal stability, with test–retest coefficients ranging from 0.696 to 0.752. Across groups, female sex, age under 60, being not at-risk-of-poverty, and no necessary medical visits during the pandemic were consistently associated with lower test–retest coefficients than their counterparts. Confirmatory factor analysis showed good model fit (CFI = 0.985, TLI = 0.975, RMSEA = 0.040, SRMR = 0.035), and measurement invariance across time points was established (ΔCFI < 0.010). Consistent with other studies, somatic symptom burdening initially decreased and subsequently increased at the third measurement time point. The increase is discussed in the context of the pandemic and the serious political crisis in 2022. Overall, the scale demonstrated good psychometric properties including sensitivity to major crises of life conditions.

**Supplementary Information:**

The online version contains supplementary material available at 10.1038/s41598-026-51808-0.

## Introduction

Somatic symptoms are reported frequently among the general population and represent more than isolated physical complaints. Prior research indicated that a higher somatic symptom load is closely linked to mental health burden, increased health care utilization, and reduced quality of life, suggesting that somatic symptoms function as an important marker of overall psychosocial vulnerability^1,2^. Somatic symptoms have characteristically been found in respondents with various medical conditions such as cancer or coronary heart disease^3,4^. Population-based studies further demonstrated that women and older adults consistently report higher somatic symptom loads, highlighting stable demographic gradients that persist beyond specific clinical contexts^1,2^. Notably, evidence also pointed to sex-specific consequences: while elevated somatic symptom burden has been associated with increased mortality risk, this association appears to be confined to men, underscoring the complex and differential health implications of somatization across sexes^1^.

Against this backdrop, findings from the SARS-CoV-2 pandemic suggested that somatic symptoms may be particularly sensitive to large-scale psychosocial stressors. Multiple studies observed a high prevalence of somatization during the pandemic, especially among individuals infected with SARS-CoV-2^5,6^ Beyond infection status, COVID-19-related anxiety was also associated with somatic symptoms^7^, alongside younger age, higher fatigue, and greater psychological symptom burden^8^. Taken together, these findings may support the notion that pandemic-related uncertainty, stress, and mental health strain likely amplified somatic symptom burden, extending established vulnerability patterns into a novel global crisis context^9^.

As somatic symptoms are common in the general population and have been found to be associated with various somatic and mental health burdens and illnesses as well as with numerous sociodemographic characteristics, it seems highly important to have short and validated instruments to screen somatic symptoms in population-based epidemiological surveys. The Somatic Symptom Scale (SSS-8) is a time-efficient 8-item tool for somatic symptom burden screening that assesses the most frequent somatic symptoms such as back, chest, and joint pain, headaches, stomach or bowel problems, dizziness, fatigue and difficulty falling asleep^10^. The scale was derived from the Patient Health Questionnaire (PHQ-15), a validated and frequently used instrument to assess somatic symptoms^11,12^. The SSS-8 has been found to have high internal consistency and validity in the past^10,13,14^.

However, data on the temporal stability of the SSS-8 are limited, and its test-retest reliability, construct validity, and temporal measurement invariance have not yet been examined in a German sample during the pandemic or across extended time periods. Assessing temporal stability and measurement invariance is crucial to ensure that observed differences reflect true change rather than measurement artefacts. Therefore, this study addresses this gap in the literature and is the first to evaluate test-retest reliability and longitudinal measurement invariance of the SSS-8 in the general population.

The large data set from the Gutenberg COVID-19 Study (GCS), collected at three separate time points during the pandemic, offers a unique opportunity to derive new and sound assessments of the temporal stability (test-retest-reliability) and construct validity of somatic complaints. The objectives of this study are to.

1. determine the trajectory of somatic symptoms over the course of the pandemic;
2. evaluate the psychometric properties of the SSS-8 including test-retest reliability and measurement invariance;
3. analyse associations with sociodemographic and psychological factors. Namely, we want to investigate whether female sex, higher age, fatigue, distress, poverty, utilization of health care services during the pandemic, lower SES, higher COVID-19-realted fear, lower quality of life, and lower subjective physical as well as mental health are positively associated with higher somatic symptom burden, in line with the discussed literature.

## Methods

### Data

Our data source was the Gutenberg COVID-19 Study (GCS), a population-based prospective cohort study, initiated in response to the outbreak of SARS-CoV-2. The GCS sample comprises of *N* = 8,121 individuals from the Gutenberg Health Study (GHS;^15^ and an additional *N* = 2,129 newly recruited individuals aged 25–44 who were enrolled specifically for the GCS. The GHS is a population-representative cohort study that aims to improve the individual risk prediction for various diseases including, cardiovascular, ophthalmological, metabolic, immune system, mental diseases, and cancer. However, because the GHS cohort has been followed over many years, its age distribution has progressively shifted toward older age groups. To ensure adequate representation of younger adults, to increase the overall representativeness of the GCS with respect to the working-age population, and to enable analyses of pandemic-related impacts in younger subgroups, an additional age-restricted subsample was recruited for the GCS.

This additional subsample was recruited using the same population-based procedures as the original GHS cohort, including random sampling from the local register’s office in the Mainz/Mainz-Bingen region with stratification by sex and place of residence. Data collection procedures and survey instruments were identical across the original GHS participants and the newly recruited GCS subsample. For the present analyses, data from both sources were pooled into a single analytic dataset, with cohort membership accounted for in sensitivity analyses where appropriate.

The baseline examination for the GCS occurred from October 2020 to April 2021 (T1), followed up from March 2021 to June 2021 (T2), and the second questionnaire-based follow-up from May 2022 to November 2022 (T3). Study participants underwent computer-assisted personal interviews (CAPI) and biomaterial sampling at the study center. To prepare for their appointments, participants received questionnaires in advance. For the current study, we only included respondents with available data at all three GCS examination time points. For interpretation and transparency purposes, we included a table in the supplementary material (Supplementary Table 1) that shows the differences between the dropouts and the final analysis sample. For this final analysis sample, we additionally excluded respondents without complete and valid SSS-8 data and those infected with SARS-CoV-2 (determined via self-report and PCR test) in order to not distort the results. This left us with a sample of *N* = 5,341 individuals.

The study followed GCP (Good Clinical Practice) and GEP (Good Epidemiological Practice) standards, the Declaration of Helsinki, and the Federal Data Protection Act, with all required ethical and data protection approvals obtained from relevant authorities.

### Measures

#### SSS-8

Somatic symptoms were assessed using the eight-item Somatic Symptom Scale (SSS-8;^13,14,16^. In this questionnaire, respondents were asked how much they were impaired by the eight most common somatic burdens such as various pains and aches, dizziness, stomach or bowel problems, feeling tired and having trouble sleeping within the past two weeks on a scale from 0 (not at all) to 4 (very much). The sum score of the SSS-8, therefore, ranges from 0 (indicating no somatic symptom burden) to 32 (indicating high somatic symptom burden).

#### Psychological Variables

Additionally, we collected data on depressive symptoms using the PHQ-9^17,18^ and generalized anxiety symptoms by the GAD-2^17–20^. Quality of life was measured using the EUROHIS-QOL 8 Item Index^23^. Fatigue was assessed only at T3 using a 4-item version of the Multidimensional Fatigue Inventory (MFI;^24^. Lastly, since the examination mostly took place during the pandemic, we inquired about COVID-19 related fears^25^, though only at T2. The fear was assessed by asking the participants to rate their agreement of seven statements on a five-point scale ranging from 0 (do not agree at all) to 4 (fully agree). These statements inquired about factors such as discomfort and fear when thinking about COVID-19, as well as having trouble sleeping or fearing to lose their life to this illness. A sum score of these items indicated the degree of fear, with higher values indicating more fear. All measures – with the exception of fatigue (T2, T3) and COVID-19 related fear (T2) – were administered at all three time points.

#### Health related variables

We inquired about subjective physical and mental health by asking the participants to rate their health status on a 4-point scale from 1 (very good) to 4 (bad). Additionally, we asked the respondents if they attended any necessary medical visits during the pandemic (yes/no).

#### Sociodemographic variables

We assessed the participants’ age, sex (assigned at birth, inquired from the population registry office), migration background, education, employment (no, irregular, part-time, fulltime current employment). We also inquired about their socioeconomic status (SES) using the index of Lampert and Kroll^26^. Additionally, we estimated if the participants are at-risk-of-poverty by using the definition of relative poverty by the European Union Statistics on Income and Living Conditions (EU-SILC^27^. We defined respondents to be at-risk-of-poverty if their net equalized income was under 60% of the median net equalized income of all households. Net equalized income was calculated by dividing the total monthly household income by a weighted household size: The first adult of a household contributed 1.0 unit to the household weight while every additional adult from an age of 14 increased the household weight by 0.5. Every child under the age of 14 increased the household weight by 0.3. The median net equalized income of 2019 was at 1790€, therefore, a net equalized income of < 1074€ was identified to be the threshold of being at-risk-of-poverty.

### Statistical procedure

Sample characteristics were computed as absolute and relative frequencies for categorical variables, while means and standard deviations were employed for continuous variables. Cohen’s d effect sizes were utilized to express mean differences, and Bonferroni-corrected t-tests were conducted to assess their significance. Internal stability of the sum scores was evaluated using Cronbach’s alpha (*α*) and McDonald’s total omega (*ω*_*t*_). Acceptable reliability is met when *α* or *ω*_*t*_ ≥ 0.70. Temporal stability of both the sum score and the individual items was determined through Pearson’s correlation coefficient. Discriminatory power coefficients were derived by calculating the corrected correlation between individual items and the total score.

Confirmatory factor analysis (CFA) was conducted to evaluate factor validity. In line with Gierk et al.^10^, we specified a higher-order general factor model comprising an overarching somatic symptom burden factor and four lower-order symptom clusters (gastrointestinal, pain, cardiopulmonary, and fatigue). Given that the SSS-8 items are ordinal and exhibited deviations from multivariate normality across all three pandemic time points, CFA and measurement invariance analyses were estimated using the weighted least squares mean- and variance-adjusted (WLSMV) estimator, which is specifically recommended for ordinal indicators and provides robust parameter estimates and fit indices under non-normality^28^. Goodness-of-fit was assessed using four criteria: the Comparative Fit Index (CFI), the Tucker Lewis Index (TLI), the Standardized Root Mean Squared Residual (SRMR), as well as the Root Mean Square Error of Approximation (RMSEA). A RMSEA and SRMR value of < 0.050 indicates a good fit while a value between 0.050 and 0.080 indicates a reasonable fit. For CFI and TLI, a value of > 0.950 demonstrates a good fit of a model^29,30^.

We conducted tests of measurement invariance across time using the sequential strategy proposed by Meredith and Teresi^31^. Our focus was exclusively on invariance between measurement time points, as the primary aim was to examine the temporal stability of the measurement model and to ensure that longitudinal changes reflected true change in somatic symptom burden rather than alterations in the measurement properties of the instrument. Accordingly, we did not test for measurement invariance across sociodemographic subgroups, as such analyses were beyond the scope of the present study. Establishing measurement invariance is a critical prerequisite for longitudinal analyses, as it ensures that the construct is measured equivalently across time points and that observed score differences can be interpreted as true changes in the underlying latent construct rather than artifacts of measurement instability. Within this framework, we evaluated configural, weak, strong, and strict invariance by progressively imposing equality constraints across models. Configural invariance represents an unrestricted baseline model, allowing factor loadings, intercepts, and residual variances to vary across time points. Weak invariance constrains factor loadings to be equal across time, strong invariance additionally constrains item intercepts, and strict invariance further assumes equality of residual variances across time points. Comparisons between increasingly constrained models allowed us to determine whether full invariance was supported.

The chi-square difference test was not used due to its sensitivity to large sample sizes and the associated risk of rejecting well-fitting models. Instead, model comparisons were based on changes in multiple approximate fit indices, including the CFI, the RMSEA, and the SRMR. Following established recommendations, invariance was considered acceptable if changes did not exceed ΔCFI ≥ 0.010, ΔRMSEA ≥ 0.015, and ΔSRMR ≥ 0.030 for tests of weak invariance or ≥ 0.010 for tests of strong and strict invariance^32–34^. Although all indices were computed, primary emphasis was placed on changes in CFI, as this index has been shown to be less sensitive to sample size and model complexity and to perform more robustly than alternative fit indices in the context of measurement invariance testing.

We explored associations with relevant sociodemographic and psychological factors through Pearson’s correlation coefficients and by examining mean score differences between groups. Age groups (< 60 vs. ≥ 60) were formed in such a way that they approximately divide the sample in half. Test-retest stability between the pandemic time points was evaluated using Pearson correlations. All analyses were conducted using R version 4.1.1 (packages: psych^35^, lsr^36^, lavaan^37^, semTools^38^.

## Results

### Sample characteristics

A sample of *N* = 5,341 respondents provided valid and complete data for all three pandemic time points (T1-T3). This included 2,750 (51.5%) women and 2,591 (48.5%) men with a mean age of 56.48 (*SD* = 14.55). More information on the sample can be seen in Table 1.

**Table 1:** Sample Characteristics at T1, *N* = 5341.

Sex (female, yes %)	Available data	Sample	α	ω_t_
	5341	2750 (51.5%)		
Age, mean (SD)	5341	56.48 (14.55)		
Age group (%)				
25–34		450 (8.4%)		
35–44		825 (15.5%)		
45–54		998 (18.7%)		
55–64		1321 (24.7%)		
65–74		1124 (21.0%)		
75+		623 (11.7%)		
Migration background (yes, %)	5336	1047 (19.6%)		
Partnership (yes, %)	4034	3659 (90.7%)		
Employment	5063			
no current employment		1650 (32.6%)		
irregularly or part-time		1235 (24.4%)		
fulltime		2178 (43.0%)		
SES, mean (SD)	5133	15.02 (4.05)		
Equalized income, mean (SD)	5073	3024.27 (1858.05)		
At-risk-of-poverty (yes, %)	5073	174 (3.4%)		
Subjective physical condition, mean (SD)	5339	1.95 (0.54)		
Subjective mental condition, mean (SD)	5339	2.01 (0.60)		
PHQ-9, mean (SD)	5186	4.27 (3.72)	0.83	0.86
GAD-2, mean (SD)	4191	0.82 (1.12)	0.75	0.75
MFI 4-item Fatigue, mean (at T2) (SD)	5252	12.07 (1.63)	0.85	0.89
Eurohis-QOL, mean (SD)	5061	32.53 (4.11)	0.83	0.86
SSS-8, mean (SD)	5341	5.70 (4.55)	0.78	0.82
Necessary medical visits during pandemic (yes)	5341	3919 (73.4%)		
COVID-19 related fear, mean (at T2) (SD)	5173	7.25 (4.75)	0.86	0.92

Note. COVID-19-related fear was only assessed at T2, while Fatigue was assessed from T2; therefore, the reported values correspond to T2 rather than T1. SES – Socioeconomic status; PHQ - Patient-Health-Questionnaire; GAD - Generalized Anxiety Disorder Screener; MFI - Multidimensional Fatigue Inventory; SSS - Somatic Symptom Scale.

### Measurement invariance

When analyzed separately, the SSS-8 score with its higher order structure yielded very good model fits for all three time points (see Table 2). Full invariance across the time points was able to be established, judging by the CFI difference which consistently stayed below the suggested threshold of 0.01. Furthermore, as strict measurement was, therefore, established, longitudinal stability as the residual level was confirmed.

**Table 2:** Confirmatory factor analysis and measurement invariance.

T1	CFI	TLI	RMSEA	SRMR	Δ CFI	Δ TLI	Δ RMSEA	Δ SRMR
	0.985	0.975	0.040	0.035				
T2	0.987	0.979	0.036	0.034				
T3	0.987	0.979	0.039	0.034				
Configural	0.987	0.978	0.039	0.031				
Metric	0.986	0.982	0.035	0.032	-0.001	0.004	-0.004	0.001
Scalar	0.985	0.982	0.035	0.033	-0.001	0.000	0.001	0.001
Strict	0.983	0.984	0.033	0.037	-0.002	0.002	0.004	0.004

Note. CFI = Robust Comparative Fit Index; TLI = Robust Tucker-Lewis Index; RMSEA = Robust Root Mean Square of Approximation; SRMR = Standardized Root Mean Square Residual; Δ = difference in model fits between sequential models.

### SSS-8 mean scores and item characteristics

The SSS-8 yielded good reliability at all three time points with Cronbach’s alpha ranging from 0.78 to 0.80 and McDonald’s omega ranging from 0.82 to 0.84. The SSS-8 mean score dipped between T1 and T2 (from *M* = 5.70, *SD* = 4.55 to *M* = 5.25, *SD* = 4.67) and subsequently increased again at T3 (*M* = 6.09, *SD* = 4.83). These changes in means were statistically significant (*p* < 0.001), however, the differences had small to negligible effect sizes with Cohen’s d ranging from 0.08 (T1-T3) to 0.18 (T2-T3). Not all items contributed significantly to the changes between time points. Between the first two time points, pain in arms, legs, or joints, dizziness, and feeling tired or having low energy were significantly different. Between T2 and T3, only suffering from headaches did not contribute significantly to the mean difference. And lastly, between T1 and T3, stomach or bowels problems, back pain, and having trouble sleeping did not yield significant differences. These results can also be seen when looking at the effect sizes of each item.

All items contributed to the SSS-8 total score at all three time points with discrimination power coefficients ranging from 0.41 to 0.64. The test-retest correlations of the SSS-8 score showed rather large coefficients with 0.752 between T1 and T2, 0.696 between T2 and T3, and 0.703 between T1 and T3. All results can be found in Table 3.

**Table 3:** SSS-8 items and score characteristics.

Item / score	T1	T2	T3	Δ_t2−t1_	Δ_t3−t2_	Δ_t3−t1_	ES_t2−t1_	ES_t3−t2_	ES_t3−t1_	*r*_it t1_	*r*_it t2_	*r*_it t3_	*r*_tt t2−t1_	*r*_tt t3−t2_	*r*_tt t3−t1_
	M (SD)	M (SD)	M (SD)	M (SD)	M (SD)	M (SD)									
SSS-8 sum score	5.70 (4.55)	5.25 (4.67)	6.09 (4.83)	-0.45*** (3.25)	0.84*** (3.71)	0.39*** (3.63)	0.10	0.18	*0.08*	*α* = 0.78 *ω* = 0.82	*α* = 0.80 *ω* = 0.84	*α* = 0.79 *ω* = 0.84	0.752	0.696	0.703
Stomach or bowel problems	0.50 (0.82)	0.39 (0.76)	0.51 (0.85)	-0.11*** (0.80)	0.12*** (0.86)	0.01 (0.87)	0.14	0.14	0.01	0.43	0.45	0.45	0.496	0.434	0.459
Back pain	1.04 (1.06)	0.95 (1.06)	1.06 (1.06)	-0.09*** (0.92)	0.10*** (1.02)	0.01 (1.01)	0.08	0.10	0.01	0.52	0.56	0.51	0.619	0.539	0.541
Pain in arms, legs, or joints	0.89 (1.03)	0.87 (1.05)	1.01 (1.07)	-0.02 (0.95)	0.14*** (1.06)	0.12*** (1.05)	0.02	0.13	0.11	0.48	0.53	0.48	0.582	0.500	0.501
Headaches	0.61 (0.87)	0.55 (0.82)	0.58 (0.85)	-0.06*** (0.76)	0.03 (0.80)	-0.03* (0.83)	0.07	0.03	0.04	0.43	0.46	0.45	0.596	0.537	0.537
Chest pain or shortness of breath	0.33 (0.69)	0.29 (0.67)	0.39 (0.77)	-0.04** (0.64)	0.10*** (0.74)	0.06*** (0.74)	0.06	0.14	0.09	0.44	0.48	0.50	0.562	0.476	0.481
Dizziness	0.30 (0.64)	0.28 (0.64)	0.38 (0.71)	-0.01 (0.61)	0.09*** (0.68)	0.08*** (0.70)	0.02	0.14	0.12	0.41	0.44	0.44	0.543	0.501	0.467
Feeling tired or having low energy	1.01 (1.01)	0.98 (1.05)	1.14 (1.08)	-0.03 (0.83)	0.16*** (0.93)	0.13*** (0.93)	0.03	0.15	0.12	0.61	0.64	0.64	0.672	0.622	0.602
Trouble sleeping	1.02 (1.07)	0.93 (1.07)	1.04 (1.09)	-0.09*** (0.86)	0.10*** (0.93)	0.02 (0.95)	0.08	0.09	0.02	0.52	0.54	0.54	0.678	0.629	0.615

Note. Δ = difference between the time points; ES = effect size of difference of the scores between the time points measured by Cohen’s *d*; r_it_ = discriminatory power calculated by corrected item-total correlations; r_tt_ = Pearson correlation coefficient for test-retest testing; α = Cronbach’s Alpha; ω = McDonald’s Omega; M = mean; SD = standard deviation; *** *p* < 0.001, ** *p* < 0.01, * *p* < 0.05.

### Associations with sociodemographic and psychological factors

Next, in Table 4, we compared the means, as well as the changes in SSS-8 scores between different sociodemographic groups. We found significant mean differences between all group comparisons at all three time points with the exception of age groups. Here we observed no significant mean difference at T2 and T3. However, this is the only group comparison that yielded significant differences in changes between the time points with the younger group (< 60) demonstrating a larger decrease between T1 and T2 as well as a larger increase between T2 and T3. In terms of temporal stability, we found rather small differences between the groups, though, women, participants under the age of 60, those not at-risk-of-poverty, as well as people without necessary medical visits during the pandemic consistently yielded lower test-retest coefficients compared to their group counterparts.

**Table 4:** Changes in SSS-8 sum score by sex, age group, being at-risk-of-poverty, and medical visits.

				Change T1-T2				Change T2-T3					
	T1	T2	T3	Δ T2-T1	decrease (%)	no change (%)	increase (%)	Δ T3-T2	decrease (%)	no change (%)	increase (%)	*r*_tt t12−t1_	*r*_tt t13−t2_
Women	6.55 (4.77)	6.08 (4.90)	6.87 (4.95)	-0.47 (3.57)	1341 (48.8%)	422 (15.3%)	987 (35.9%)	0.80 (3.99)	896 (32.6%)	406 (14.8%)	1448 (52.6%)	0.727	0.673
Men	4.80 (4.13)	4.38 (4.25)	5.27 (4.55)	-0.42 (2.87)	1173 (45.3%)	588 (22.7%)	830 (32.0%)	0.89 (3.39)	752 (29.0%)	480 (18.5%)	1359 (52.5%)	0.765	0.706
*Group difference* *(p-value)*	***0.000***	***0.000***	***0.000***	*0.548*				*0.374*					
< 60	5.82 (4.59)	5.16 (4.64)	6.17 (4.87)	-0.66 (3.48)	1517 (49.6%)	568 (18.6%)	972 (31.8%)	1.02 (4.03)	915 (29.9%)	489 (16.0%)	1653 (54.1%)	0.716	0.642
≥ 60	5.55 (4.50)	5.38 (4.72)	5.98 (4.76)	-0.17 (2.90)	997 (43.6%)	442 (19.4%)	845 (37.0%)	0.60 (3.21)	733 (32.1%)	397 (17.4%)	1154 (50.5%)	0.804	0.771
*Group difference* *(p-value)*	***0.032***	*0.087*	*0.150*	***0.000***				***0.000***					
At-risk-of-poverty	6.68 (5.19)	6.17 (5.56)	6.93 (5.11)	-0.51 (3.37)	83 (47.7%)	33 (19.0%)	58 (33.3%)	0.76 (3.70)	51 (29.3%)	29 (16.7%)	94 (54.0%)	0.805	0.763
Not at-risk	5.61 (4.49)	5.18 (4.61)	6.01 (4.76)	-0.43 (3.22)	2299 (46.9%)	936 (19.1%)	1664 (34.0%)	0.83 (3.68)	1510 (30.8%)	817 (16.7%)	2572 (52.5%)	0.750	0.693
*Group difference (p-value)*	***0.002***	***0.006***	***0.013***	*0.747*				*0.803*					
No medical visits during pandemic	4.63 (4.02)	4.22 (40.6)	5.18 (4.46)	-0.41 (3.06)	651 (45.8%)	305 (21.4%)	466 (32.8%)	0.96 (3.60)	395 (27.8%)	274 (19.2%)	753 (53.0%)	0.713	0.646
Medical visits during pandemic	6.09 (4.67)	5.63 (4.83)	6.42 (4.91)	-0.46 (3.32)	1863 (47.5%)	705 (18.0%)	1351 (34.5%)	0.80 (3.74)	1253 (92.0%)	912 (15.6%)	2054 (52.4%)	0.756	0.705
*Group difference (p-value)*	***0.000***	***0.000***	***0.000***	*0.584*				*0.157*					

Note. r_tt_ = Pearson correlation coefficient for test-retest testing.

Following these group comparisons, we evaluated the associations with the SSS-8 sum score further by correlating the score with relevant sociodemographic and psychological factors (see Table 5). Depressiveness, generalized anxiety, fatigue, female sex, being at-risk-of-poverty, subjective physical as well as subjective mental health were consistently found to have positive associations with the SSS-8. The Pearson correlation coefficients ranged from 0.035 (being at-risk-of-poverty) to 0.692 (PHQ-9). Quality of life and SES showed a significant negative association with the SSS-8 with Pearson’s correlation coefficients ranging from − 0.142 (SES) to -0.588 (QOL). Interestingly, attending necessary medical visits during the pandemic was initially associated negatively with SSS-8 at T1 and subsequently positively at T2 and T3.

**Table 5:** Pearson correlation of the SSS-8 sum score at with relevant factors.

PHQ-9	SSS-8 at T1	SSS-8 at T2	SSS-8 at T3
	0.679*** [0.673–0.702]	0.692*** [0.677–0.706]	0.690*** [0.676–0.704]
GAD2	0.417*** [0.394–0.439]	0.527*** [0.507–0.547]	0.512*** [0.492–0.532]
Eurohis-QOL	-0.550*** [-0.569 - -0.531]	-0.570*** [-0.588 – -0.551]	-0.588*** [-0.605 – -0.570]
Fatigue	n/a	0.110*** [0.084–0.137]	0.153*** [0.126–0.179]
Age (continuous)	-0.025 [-0,05–0.001]	0.042** [0.015–0.068]	0.000 [-0.026–0.027]
Female sex	0.192*** [0.166–0.218]	0.181*** [0.155–0.207]	0.166*** [0.140–0.192]
At-risk-of-poverty	0.043** [0.016–0.071]	0.039** [0.011–0.066]	0.035* [0.008–0.062]
SES	-0.142*** [-0.168 – -0.115]	-0.154*** [-0.181 – -0.127]	-0.145*** [-0.172 – -0.118]
Medical visits necessary during pandemic (yes)	-0.142*** [-0.168 – -0.115]	0.130*** [0.103–0.156]	0.096*** [0.068–0.122]
COVID-19 related fear	n/a	0.307*** [0.282–0.331]	n/a
Subjective physical health	0.398*** [0.376–0.421]	0.470*** [0.448–0.490]	0.572*** [0.553–0.590]
Subjective mental health	0.396*** [0.373–0.418]	0.429*** [0.407–0.451]	0.503*** [0.483–0.524]

Note. COVID-19-related fear was only assessed at T2, while Fatigue was assessed from T2; therefore, correlations were only reported for these two measurement time points. PHQ - Patient-Health-Questionnaire; GAD - Generalized Anxiety Disorder Screener; QOL – Quality of Life; SES - Socioeconomic status. *** *p* < 0.001, ** *p* < 0.01, * *p* < 0.05.

## Discussion

As the COVID-19 pandemic and the measures implemented to contain its spread introduced new physical and mental health challenges, they also created a unique opportunity to examine changes in somatic symptom burden. The SSS-8 has been shown to provide a rapid and reliable assessment of somatic symptom burden, making it well suited for monitoring both the general population and at-risk groups. The present study evaluated the stability of the SSS-8 and its associations with demographic and psychological factors under pandemic conditions. To this end, we used data from three measurement time points across different phases of the pandemic to examine changes in somatic symptom burden over time, which constituted the first aim of this study.

We found a significant decrease from the first to the second time point and a significant subsequent increase to the third time point. The effect sizes of these changes, however, were small to negligible with a Cohen’s d of 0.10 between T1 and T2 and d = 0.18 between T2 and T3. The score means of our data were higher than those reported by Gierk et al.^10^; *M* = 3.23, *SD* = 3.96), with means in our sample ranging from 5.25 (*SD* = 4.67) to 6.09 (*SD* = 4.83). These differences may reflect contextual factors related to data collection during the COVID-19 pandemic, as well as methodological differences between our regional panel and the nationally representative German sample used in the validation study.

We also found a drop in somatic symptom burdens between the first and the second time point, which is consistent with previous findings suggesting an initial adjustment to the pandemic restrictions^39^. This finding also aligns with theoretical models of stress adaptation, in which an initial increase in distress is followed by partial habituation once individuals adjust to new environmental demands. However, the initial decrease was followed by subsequent increase at the third measurement time point. While this rise may partly reflect the continuation of pandemic-related restrictions (which were only fully lifted in April 2023), it is also noteworthy that the third wave of data collection in 2022 coincided with the Russian invasion of Ukraine in February 2022. Prior German^40^ and European studies^41^ have reported increased psychological distress during this period, attributed to factors such as renewed refugee movements, concerns about energy security, geopolitical instability, and perceived threats to personal safety. Taken together, these contextual factors may have contributed to the observed increase in somatic symptoms in 2022, which exceeded both pandemic and pre-pandemic levels. This pattern was also evident at the item level, with increases observed for multiple pain-related symptoms (back, stomach, arms, legs, joints, and chest) as well as fatigue and sleep problems between T2 and T3. These parallel increases across several symptom domains suggest a broad somatic stress response and may reflect the cumulative impact of the prolonged psychosocial strain of the pandemic (lockdowns, home office, care burden) on physical health, rather than isolated or condition-specific effects.

The next objective of this study was to investigate the psychometric properties of the SSS-8. Internal consistency was good across all measurement points, with Cronbach’s alpha ranging from 0.78 to 0.80 and McDonald’s omega ranging from 0.82 to 0.84, consistent with previous findings^10,13^. Importantly, test–retest correlation coefficients were high (*r*_*tt*_ = 0.696 to 0.752), indicating substantial temporal stability of SSS-8 scores over time. To our knowledge, this is the first study to directly examine the test–retest reliability of the SSS-8, precluding direct comparison with prior research. In addition, we tested measurement invariance across the measurement time points and were able to establish full invariance. Together, these findings indicate that the SSS-8 reliably captures stable individual differences in somatic symptom burden over time and that observed changes are unlikely to reflect measurement artefacts. This temporal stability is particularly relevant for longitudinal epidemiological monitoring, as it supports the use of the SSS-8 to track population-level changes in somatic symptoms across prolonged stressors or societal crises. In this context, the SSS-8 may serve as a robust tool for public health surveillance and for informing health policy decisions by enabling reliable comparisons across time and critical events. Finally for the psychometric evaluation, our CFA confirmed the higher-order general-factor model which organizes the eight somatic symptoms into four clusters (gastrointestinal, pain, fatigue, and cardiopulmonary aspects), in line with previous conceptualizations^10,13^.

As a third aim of the study, we investigated the associations with sociodemographic and other relevant factors. To do this, we first compared the SSS-8 score means and changes between different sociodemographic groups. We found significant mean differences in all groups at all measurement time points except for age. Here, only the means at T1 were significantly higher for people younger than 60 than for those older than 60. This might be due to the heightened psychosocial stress that younger people faced during lockdown impairing education, social exchange and childcare particularly in the younger generations^39^. Additionally, women, people at-risk-of-poverty and respondents who had necessary medical visits during the pandemic consistently reported higher somatic symptom burdens. These findings are in line with previous results from Kliem et al.^13^ and Gierk et al.^10^ who found women, younger people and people with health care visits in the past 12 months had higher SSS-8 scores. The temporal stability was higher among men, people over the age of 60, people at-risk-of-poverty, and those with necessary medical visits during the pandemic. These findings highlight the importance of not only describing the level of somatic symptom burdening but also its stability. For instance, high stability among those at-risk-of-poverty may indicate that it is challenging for economically disadvantaged individuals who were at a high symptom level to ‘bounce back’ after an increase of somatic symptoms. Given the restrictions on medical visits during the pandemic, the need for medical visits may also be an indicator of vulnerability.

Next, we analyzed the correlations between sociodemographic and other relevant factors. Depressiveness, generalized anxiety, fatigue, female sex, being at-risk-of-poverty, having necessary medical visits during the pandemic, COVID-19 related fear, as well as subjective physical and mental health were positively associated with SSS-8, while quality of life and SES were negatively correlated. These findings are in line with results from previous studies^7–10,13^ and therefore indicate construct validity. The only factor not significantly associated was age. In previous surveys^1,2,10,42^, higher symptom load was consistently associated with female sex and higher age. Thus, it is surprising that younger respondents under 60 scored higher during the first wave of the pandemic than the elderly participants. As we had shown previously^39^, young participants were more strongly affected by the pandemic restrictions than elderly participants. Similarly, another study conducted during the pandemic came to the conclusion that younger age was associated with higher symptomatic burdens^8,43^. Perhaps these two effect directions cancelled each other out resulting in a non-significant association. This pattern suggests that contextual stressors may temporarily override typical age-related symptom distributions, underscoring the importance of situating epidemiological findings within broader societal and theoretical frameworks. Finally, we found that the direction of the association between necessary medical visits and somatic symptom burden varied across pandemic phases, even though symptom load was higher among those who attended medical visits through the pandemic. The observed change in the direction of the association may be attributable to the context at the first measurement time point, when infection incidence rates were very high and a strict lockdown was in place. Individuals with a high symptom burden are likely to have underlying chronic health conditions, placing them at greater risk for severe complications during and after infection. This heightened vulnerability may have discouraged them from attending medical visits due to fear of potential health risks, resulting in a negative association between somatic symptom burden and healthcare utilization during the initial phase. As infection rates declined over time, this fear may have diminished, leading individuals to resume medical visits. These findings underscore the context-dependent nature of risk and protective factors during prolonged public health crises.

### Strengths and limitations

Although the availability of three pandemic-related measurement time points represents a major strength, the final analytic sample was relatively older (mean age = 56.48, SD = 14.55) and socioeconomically more advantaged, with only 3.4% of participants classified as at risk of poverty, compared with 14.7% in the general German population^44^. An older and wealthier sample may underestimate overall somatic symptom burden, as younger individuals and those with lower socioeconomic status tend to report higher symptom levels and greater health-related vulnerability. These discrepancies likely reflect selective attrition across waves. As shown in Supplementary Table 1, participants who dropped out were younger and more likely to have a migration background, lower socioeconomic status, lower income, and to be at risk of poverty. Such attrition effects reduce the representativeness of the sample and may bias longitudinal estimates, warranting cautious interpretation of the findings.

Moreover, attrition may have been nonrandom with respect to health status. Individuals with higher mental or physical symptom burden may have been less able or willing to participate in follow-up assessments, potentially leading to an underestimation of symptom trajectories over time. In addition, it should be noted that a COVID-19 infection may have contributed to a heightened symptom load at T3. Consequently, the exclusion of participants with prior COVID-19 infection, while intended to reduce confounding, may have resulted in a lower reported symptom burden at T3 in our sample. Thus, although this exclusion criterion may have partially mitigated certain biases, it cannot fully compensate for differential loss to follow-up or the potential impact of infection-related symptom exacerbation. To address these limitations and to further underscore the robustness of our findings, Tables 2, 3, 4 and 5 were calculated using the full T1 sample. These tables, which can be found in the Supplementary Material, provide additional context and support for the reported associations.

Finally, it should be noted that the SSS-8 may vary in its sensitivity to changes in specific somatic symptoms across demographic and socioeconomic subgroups; future research could address this issue using latent growth modeling or multi-group confirmatory factor analysis.

## Conclusion

The SSS-8 was found to be a reliable instrument for assessing somatic symptom burden in a longitudinal setting. It demonstrated good temporal stability and sensitivity to external contextual factors such as the onset of the COVID-19 pandemic. Importantly, the establishment of longitudinal measurement invariance indicates that the SSS-8 assesses somatic symptoms consistently over time, ensuring that observed changes reflect true changes in symptom burden rather than measurement artefacts. Thus, the SSS-8 can be considered psychometrically robust for use in longitudinal population-based research, allowing for meaningful comparability of somatic symptom assessments across time. From a practical perspective, this robustness supports the use of the SSS-8 in public health surveillance and epidemiological monitoring to identify vulnerable subgroups, track symptom trajectories during societal crises, and inform targeted prevention and health policy interventions.

## Electronic Supplementary Material

Below is the link to the electronic supplementary material.

Supplementary Material 1

## Data Availability

Written consent from GCS study participants does not allow public access to the data. Access to the data in the local database is possible at any time upon request according to the ethics vote. This concept was developed with the local data protection officer and the ethics committee (local ethics committee of the Rhineland-Palatinate Medical Association, German). Interested scientists can make their requests to the Gutenberg COVID-19 Study Steering Committee (E-Mail: info-gcs@unimedizin-mainz.de).
