# Supplementary material for: Longitudinal evaluation of the psychometric stability of the somatic symptom scale-8 (SSS-8) in a large German general population sample
Source: Sci Rep. 2026 May 21;16:15872. doi: 10.1038/s41598-026-51808-0 (PMC13194760; doi:10.1038/s41598-026-51808-0)
Supplement: Supplementary file 1 — Supplementary Material 1 [file 41598_2026_51808_MOESM1_ESM.docx]

Supplementary material

**Longitudinal evaluation of the psychometric stability of the Somatic Symptom Scale-8 (SSS-8) in a large German general population sample**

**Supplementary Table 1.** Comparison of baseline characteristics between respondents dropping out between BL (T1) and the second follow-up (T3) and the final analysis

|  | **Dropouts** | **Analysis sample** |
| --- | --- | --- |
|  | *N* = 3,777 | *N* = 5,341 |
| Sex (female, yes %) | 1,871 (49.6%) | 2,750 (51.5%) |
| Age, mean (SD) | 53.58 (16.98) | 56.48 (14.55) |
| Age group (%) |  |  |
| 25-34 | 623 (16.5%) | 450 (8.4%) |
| 35-44 | 759 (20.1%) | 825 (15.5%) |
| 45-54 | 598 (15.8%) | 998 (18.7%) |
| 55-64 | 655 (17.4%) | 1,321 (24.7%) |
| 65-74 | 551 (14.6%) | 1,124 (21.0%) |
| 75+ | 589 (15.6%) | 623 (11.7%) |
| Migration background (yes, %) | 892 (24.7%) | 1,047 (19.6%) |
| Partnership (yes, %) | 2,115 (88.0%) | 3,659 (90.7%) |
| Employment |  |  |
| no current employment | 933 (28.8%) | 1,650 (32.6%) |
| irregularly or part-time | 779 (24.1%) | 1,235 (24.4%) |
| fulltime | 1,522 (47.1%) | 2,178 (43.0%) |
| SES, mean (SD) | 14.19 (4.36) | 15.02 (4.05) |
| Equalized income, mean (SD) | 2,817.53 (1,846.12) | 3,024.27 (1,858.05) |
| At-risk-of-poverty (yes, %) | 195 (6.2%) | 174 (3.4%) |
|  |  |  |
| Subjective physical condition, mean (SD) | 2.02 (0.60) | 1.95 (0.54) |
| Subjective mental condition, mean (SD) | 2.08 (0.63) | 2.01 (0.60) |
| PHQ-9, mean (SD) | 5.01 (4.27) | 4.27 (3.72) |
| GAD-2, mean (SD) | 0.97 (1.26) | 0.82 (1.12) |
| Eurohis-QOL, mean (SD) | 31.28 (4.56) | 32.53 (4.11) |
| SSS-8, mean (SD) | 6.54 (5.16) | 5.70 (4.55) |
|  |  |  |
| Necessary medical visits during pandemic (yes) | 2.408 (68.4%) | 3,919 (73.4%) |

The following supplementary Tables 2 – 5 are calculated using the full sample of T1 to further underscore the robustness of our results.

**Supplementary Table 2.** SSS-8 items and score characteristics for the T1 sample.

| **Item / score** | **T1** | **Internal stability** |
| --- | --- | --- |
|  | *M (SD)* |  |
| SSS-8 sum score | 6.01 (4.80) | *α* = 0.79  *ω* = 0.83 |
|  |  |  |
| Stomach or bowel problems | 0.53 (0.86) |  |
| Back pain | 1.11 (1.09) |  |
| Pain in arms, legs, or joints | 0.94 (1.07) |  |
| Headaches | 0.65 (0.91) |  |
| Chest pain or shortness of breath | 0.37 (0.75) |  |
| Dizziness | 0.33 (0.69) |  |
| Feeling tired or having low energy | 1.09 (1.05) |  |
| Trouble sleeping | 1.04 (1.10) |  |

**Supplementary Table 3.** Confirmatory factor analysis for the T1 sample

|  | **CFI** | **TLI** | **RMSEA** | **SRMR** |
| --- | --- | --- | --- | --- |
| T1 | 0.985 | 0.976 | 0.040 | 0.035 |

**Supplementary Table 4.** Changes in SSS-8 sum score by sex, age group, being at-risk-of-poverty, and medical visits for the T1 sample

|  | **T1** |
| --- | --- |
| Women | 6.94 (5.07) |
| Men | 5.03 (4.29) |
| *Group difference (p-value)* | ***<.001*** |
|  |  |
| < 60 | 6.13 (4.86) |
| ≥ 60 | 5.82 (4.71) |
| *Group difference (p-value)* | ***0.002*** |
|  |  |
| At-risk-of-poverty | 7.20 (5.58) |
| Not at-risk | 5.90 (4.75) |
| *Group difference (p-value)* | ***<.001*** |
|  |  |
| No medical visits during pandemic | 5.12 (4.49) |
| Medical visits during pandemic | 6.36 (4.88) |
| *Group difference (p-value)* | ***<.001*** |

**Supplementary Table 5.** Pearson correlation of the SSS-8 sum score at T1 with relevant factors

|  | **SSS-8 at T1** |
| --- | --- |
| PHQ-9 | 0.689*** |
| GAD2 | 0.527*** |
| Eurohis-QOL | -0.557*** |
| Age (continuous) | -0.024* |
| Female sex | 0.199*** |
| At-risk-of-poverty | 0.055*** |
| SES | -0.150*** |
| Medical visits necessary during pandemic (yes) | -0.117*** |
| Subjective physical health | 0.423*** |
| Subjective mental health | 0.400*** |

*Note*. PHQ - Patient-Health-Questionnaire; GAD - Generalized Anxiety Disorder Screener; QOL – Quality of Life; SES - Socioeconomic status. *** p < 0.001, ** p < 0.01, * p < 0.05.
